# Discriminating imaging findings of acute osteoporotic vertebral fracture: a prospective multicenter cohort study

**DOI:** 10.1186/s13018-014-0096-1

**Published:** 2014-10-10

**Authors:** Khalid Mohammad Qasem, Akinobu Suzuki, Kentaro Yamada, Masatoshi Hoshino, Tadao Tsujio, Shinji Takahashi, Hiroaki Nakamura

**Affiliations:** Department of Orthopaedic Surgery, Osaka City University Graduate School of Medicine, 1-4-3 Asahi-machi, Abeno-ku, Osaka, 545-8585 Japan; Spine Center, Shiraniwa Hospital, 6-10-1 Shiraniwadai, Ikoma, Nara 630-0136 Japan

**Keywords:** Osteoporosis, Vertebral fracture, Plain X-ray, Magnetic resonance imaging, Chronological change

## Abstract

**Background:**

Appropriate treatment of osteoporotic vertebral fractures (OVF) requires knowledge of the age of the fracture. Although diagnostic imaging has made remarkable progress in recent years, it remains difficult to differentiate acute fractures from old. Our purpose was to investigate chronological changes in radiological findings after OVF and to identify discriminators of acute versus older injuries.

**Methods:**

We evaluated 139 vertebrae in 136 patients. All patients underwent X-ray and magnetic resonance imaging (MRI) examination within 2 weeks of injury and again after 6 months. The anterior vertebral height ratio (AVHR) was calculated on lateral X-ray, and the intensity change of the posterior wall of the fractured vertebra was evaluated on T1-weighted MRI. The cutoff AVHR value to diagnose acute fracture was determined by receiver operating characteristic (ROC) curve analysis.

**Results:**

Average AVHR fell from 84.6% at initial visit to 63.7% at 6 months. When acute fracture was defined as AVHR >75%, sensitivity was 85.6%, specificity was 67.6%, and positive predictive value was 72.6%. On MRI, 83.5% of fractured vertebrae showed intensity change in the posterior wall in the acute stage, which fell to 41.7% of vertebrae after 6 months. When intensity change in the posterior wall and AVHR >75% were both present, the specificity and positive predictive value for diagnosing acute fracture improved to 87.1% and 84.7%, respectively.

**Conclusions:**

This study suggests that vertebral fracture rarely shows significant collapse on X-ray in the first 2 weeks after injury. The combination of intensity change in the posterior wall on MRI and AVHR >75% on X-ray indicates a high probability of acute fracture.

## Background

The percentage of the population over 65 years of age is increasing in developed countries. In line with this trend, the number of patients with osteoporosis is also increasing [1,2]. The most common type of fracture associated with osteoporosis is osteoporotic vertebral fracture (OVF). Because OVF has a large adverse effect on activities of daily living and quality of life [3,4], appropriate treatment is essential at each stage of injury. In the early stages, conservative treatment is often used. Bracing or patient education for daily activity should be started immediately, because delaying these therapeutic interventions may slow healing or may lead to severe vertebral collapse, affecting the patient’s future ability in activities of daily living and impairing quality of life [5-7]. Delayed union and pseudarthrosis after OVF can cause prolonged back pain and progressive collapse of the fractured vertebral body [8]. Kyphosis-induced worsening of truncal balance and collapse of vertebral bodies have been cited as major causes of decreased quality of life in elderly patients [7]. For cases resistant to conservative treatment, alternative options such as vertebroplasty, kyphoplasty, and/or other surgical treatments such as osteotomy may be applied to stabilize the fractured vertebrae [9] or to correct alignment [10]. Because the choice of treatment varies with the stage of the fracture, it is important to know the duration after injury to select the appropriate treatment option. OVF is usually diagnosed on plain X-ray, but it is often difficult to distinguish acute vertebral injury on plain films because of various vertebral deformities, including old fractures. Many studies have shown the usefulness of MRI in conjunction with plain X-ray in diagnosing OVF [11-13]. However, few reports have described the time-dependent changes in imaging findings on both plain radiographs and MRI after OVF, and the distinguishing features of acute fracture remain unknown.

In this study, we followed 136 patients with OVF for 6 months and investigated radiological changes over time. The purpose of this study was to evaluate the changes in vertebral height ratio on X-ray and intensity change on T1-weighted MR images and to identify indicators of acute versus chronic vertebral fractures associated with osteoporosis.

## Methods

### Patient selection

Twenty-five institutions in the Osaka area of Japan participated in this prospective cohort study [14,15]. Patients older than 65 years with recent OVFs were enrolled and provided informed consent. All patients were examined with plain radiographs and MRI of the spine, and orthopedic surgeons at each institution diagnosed vertebral fracture based on acute back pain with abnormal radiological findings. Pathological fractures associated with tumors were excluded. The study was preapproved by the Ethical Committees for Clinical Research at the respective institutions. Between June 2005 and September 2007, a total of 485 patients were enrolled. At the time of enrollment, all patients were treated conservatively. Treatment decisions, including type of brace, duration of bracing, and medications, were made by individual doctors based on their experience. Among the 485 patients, 15 died, 11 were excluded because of other diseases (e.g., heart failure, cerebral infarction, pulmonary emphysema), and 39 were lost to follow-up. In all, 420 patients completed the 6-month follow-up (86.6% follow-up rate).

In this study, we included only those patients whose injury date was identified by pain onset or specific known injury. Patients whose MRI was not examined within 2 weeks after injury, whose MRI was not examined at 6-month follow-up, and/or whose image quality was poor were excluded. Patients with additional acute or chronic fracture of adjacent vertebrae were also excluded because this could complicate comparison of the shape and color of the targeted acutely injured vertebrae with adjacent vertebrae. Overall, 139 vertebrae in 136 patients (21 men and 126 women) were analyzed in this study.

### Imaging analysis

**Figure 1:**
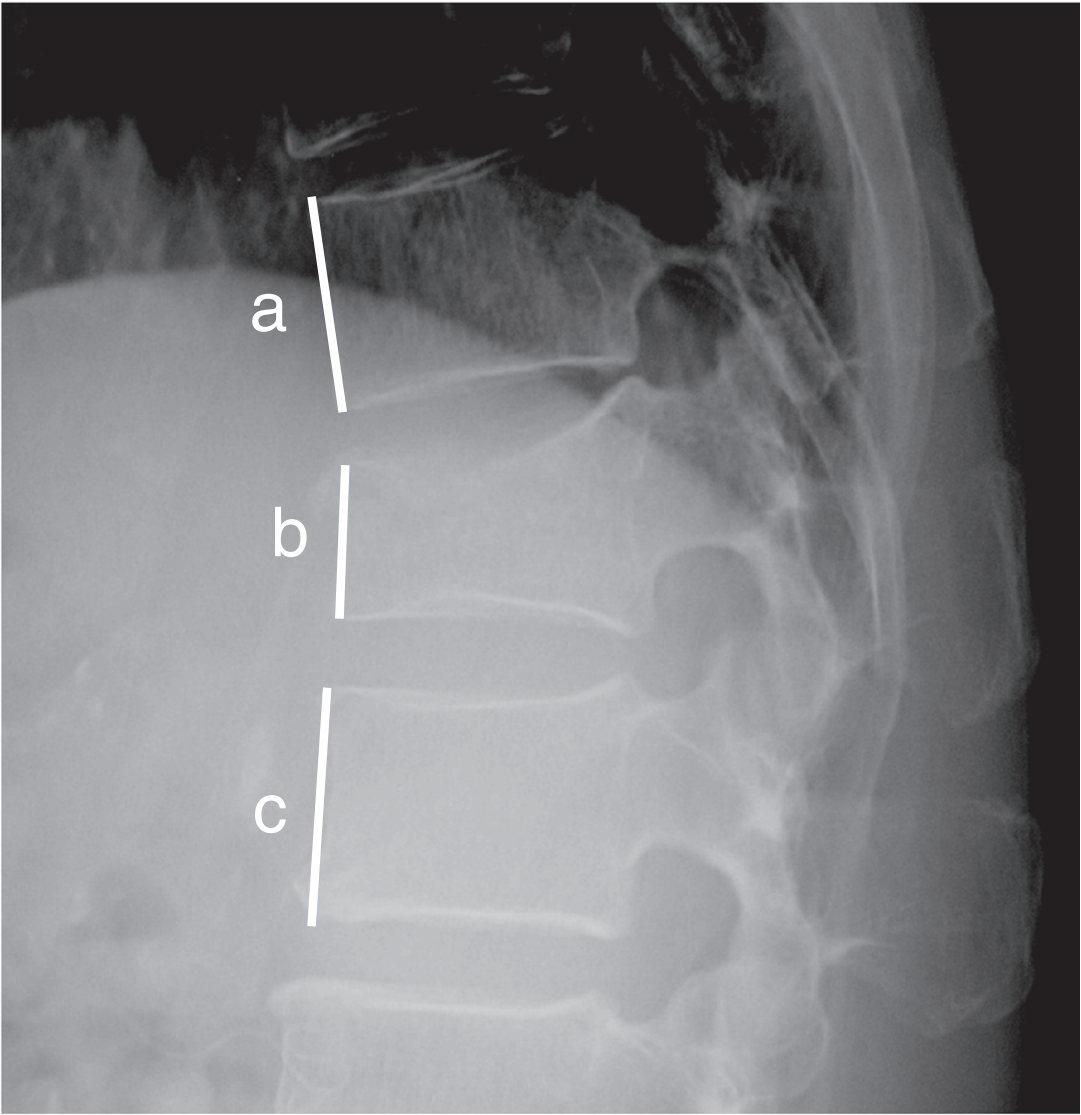
**Anterior vertebral height ratio =2b / (a + c) × 100 (%).**

Plain X-rays were taken with patients in the lateral decubitus position. We calculated the anterior vertebral height ratio (AVHR) as the anterior body height of the fractured vertebra divided by the height of the adjacent intact vertebral body (Figure 1) [14,15] at the time of enrollment and at 6-month follow-up. Non-union at 6 months was diagnosed if there was instability (change in shape with vertebral cleft) of the fractured vertebra between flexed and extended positions on plain radiographs.

**Figure 2:**
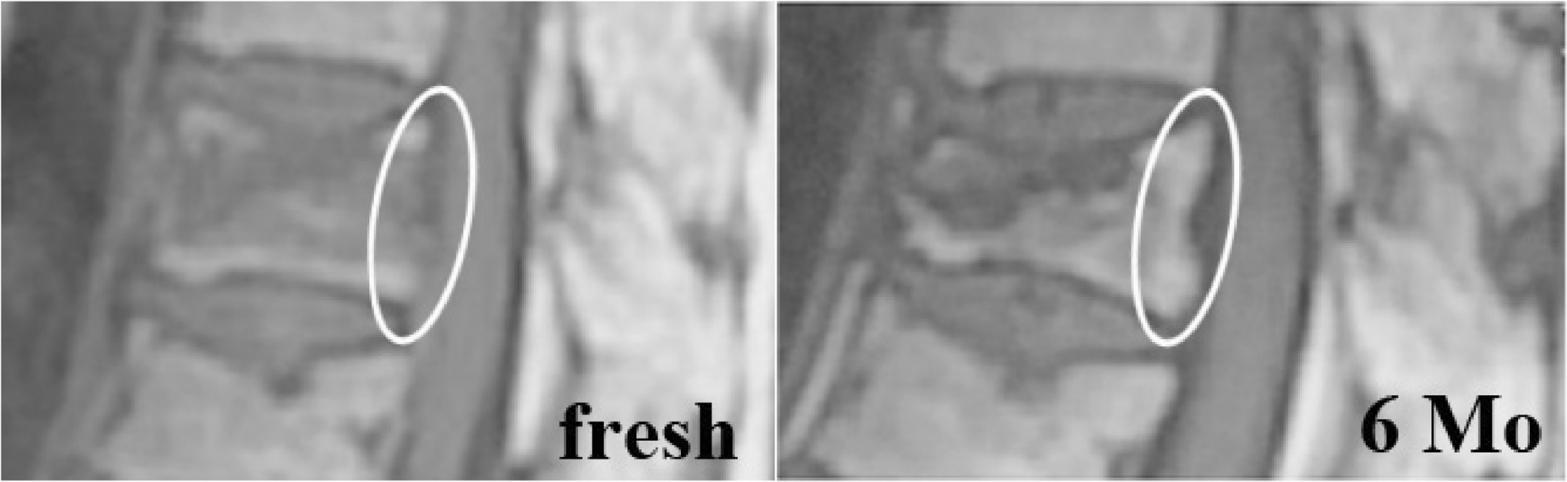
**Representative image of chronological intensity change at posterior wall on T1-weighted magnetic resonance image.** Intensity change at posterior wall was observed in the acute stage (left), but the intensity change was disappeared at 6 months (right).

Three experienced orthopedic surgeons who were not provided with patient information evaluated the MRI images. Change in signal intensity at the posterior wall of fractured vertebrae was evaluated on T1-weighted images (Figure 2a, b) and recorded as positive or negative. The three surgeons initially evaluated all MRI images independently. The evaluations were consistent for 201 of 278 images (72.3%). For the remaining images without consensus, the three doctors discussed the cases and together determined the final evaluation. In this study, we did not include the evaluation of T2-weighted images because intensity on these images varies too much between patients to achieve agreement on the evaluation [12].

### Statistical analysis

Data are presented as mean ± SD. Sensitivities and specificities for the best cutoff value of AVHR to diagnose acute fractures versus old fractures were calculated with receiver operating characteristic (ROC) curve analysis using a computer software (SPSS version 19.0; IBM Co., NY, USA).

## Results

Patient demographic data are shown in Table 1. The mean patient age was 75.9 ± 6.6 years (range, 65–91 years). Fractures were most common at L1 (35.3%), followed by T12 (26.6%) and L2 (10.8%). All patients were treated conservatively during the follow-up period.

**Table 1:** **Demographic data**

**Demographic data**	**Number**
Sex (female), *n* (%)	116 (85.3)
Age, mean ± SD, (range), years	75.9 ± 6.6 (65–91)
Level, *n*	
T6	1
T7	1
T8	1
T9	5
T10	1
T11	10
T12	37
L1	49
L2	15
L3	11
L4	8
Period to first X-ray examination from injury, mean ± SD, (range), days	3.2 ± 3.5 (0–14)
Period to first MRI examination from injury, mean ± SD, (range), days	6.2 ± 3.9 (0–14)
Hospitalization, *n* (%)	97 (70.8)
Orthosis	
Tailor-made hard corsets, *n* (%)	27 (19.7)
Tailor-made elastic corsets, *n* (%)	69 (50.4)
Ready-made elastic corsets, *n* (%)	26 (19.0)
None	14 (10.2)
Non-union (6 months), *n* (%)	15 (10.8)

### Chronological change in anterior vertebral height ratio on plain X-ray

**Figure 3:**
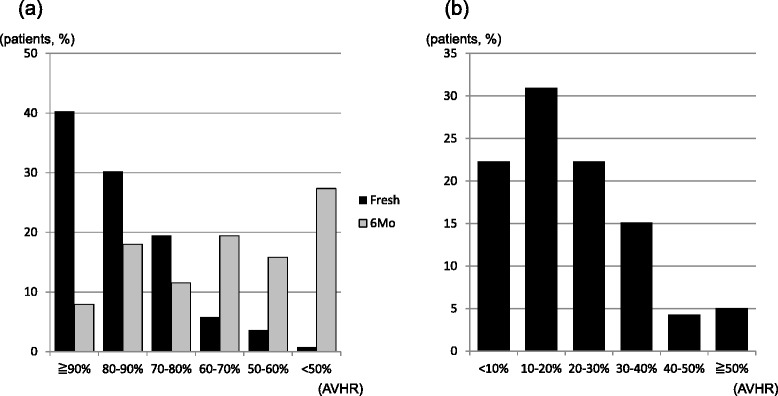
**Patient distribution of anterior vertebral height ratio (AVHR) on plain X-ray. (a)** Patient distribution of AVHR within 2 weeks of injury (acute) and at 6 months after injury (old). **(b)** Change in AVHR between the two time points.

On plain X-ray within 2 weeks after injury, the mean AVHR was 84.6 ± 11.7%. One hundred twenty-five vertebrae (90%) with acute OVF had AVHR greater than 70% (Figure 3a). At 6-month follow-up, the mean AVHR decreased to 63.7 ± 11.7%, and 65.2% of all vertebrae had AVHR less than 70% (Figure 3a). By 6 months after injury, 77.7% of vertebral fractures had more than 10% further collapse than at initial evaluation, and 24.5% of vertebrae had collapsed more than 30% further (Figure 3b).

### Chronological change in intensity at posterior wall on MRI

On T1-weighted MRI, 116 vertebrae (83.5%) showed a low-intensity change at the posterior wall of the fractured vertebra in the acute stage, and 58 (41.7%) had a persistent hypointensity after 6 months. Among the vertebrae with low-intensity change in the acute stage, signal intensity had normalized by 6 months in 65 vertebrae (56%).

### Sensitivity, specificity, and positive predictive value for the diagnosis of acute OVF

**Figure 4:**
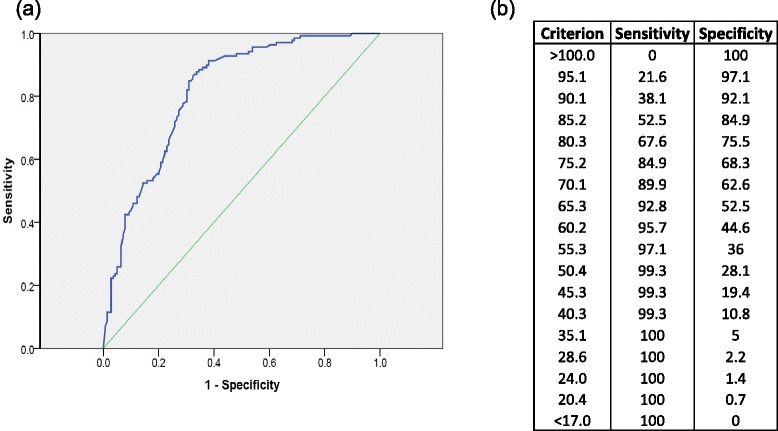
**Receiver operating characteristics (ROC) curve for discrimination of acute osteoporotic vertebral fracture from older fracture. (a)** ROC curve for the discrimination of acute osteoporotic vertebral fracture from older fracture based on anterior vertebral collapse ratio, and **(b)** sensitivity and specificity for various cutoff criteria.

The area under the curve (AUC) for the ROC (Figure 4) was 0.815 (95% CI, 0.765–0.865), indicating good discrimination of acute OVF from older OVF based on the AVHR on plain X-ray. A cutoff value of >75% for the AVHR gave optimal sensitivity and specificity for diagnosing acute versus older OVF (sensitivity 85.6%, specificity 67.6%, positive predictive value (PPV) 72.6%, negative predictive value (NPV) 82.4%). When acute fracture was diagnosed based on low-intensity change at the posterior wall on T1-weighted images, sensitivity was 83.5%, specificity was 58.3%, PPV was 66.7%, and NPV was 77.9%. When both AVHR >75% and intensity change were present, sensitivity decreased to 71.9% and NPV to 75.7%, but the specificity and PPV markedly improved to 87.1% and 84.7%, respectively.

### The relationship between posterior wall hypointensity and non-union at 6 months

At 6-month follow-up, 14 vertebrae (10.1%) were diagnosed with non-union. Among these, 7 did not show low-intensity change at the posterior wall on T1-weighted images at 6 months, although some intensity change was found on other parts of the vertebra or on the T2-weighted image. Using low-intensity change at the posterior wall on T1-weighted images to diagnose non-union at 6 months, sensitivity, specificity, and positive predictive value were 50.0%, 59.2%, and 12.1%, respectively.

## Discussion

In this study, we included only patients who underwent imaging studies within 2 weeks after injury. We attempted to clarify the characteristics of acute vertebral fracture that differentiate it from older fractures by comparing radiological images from the first 2 weeks with those obtained 6 months after injury. Several studies have focused on the diagnosis of OVF, but many of these aimed to differentiate acute OVF from malignant fracture or osteomyelitis [11,16,17]. To our knowledge, this is the first study to focus on the differential diagnosis of acute versus old fracture.

Because X-ray is the simplest and most common test used to diagnose OVF, many studies have been performed to establish diagnostic methods using plain films [18,19]. Genant et al. reported a semi-quantitative method that assessed OVF by visual determination of the extent of vertebral height reduction and morphological change [18]. This semi-quantitative method standardizes the evaluation of apparent change in vertebral dimensions, so that a vertebral fracture is identified if vertebral height is more than 20%–25% less than expected. This assessment is intelligible and easy to use; however, this and other quantitative methods may not be sufficient in diagnosing acute fractures. Pham et al. reported that 21 vertebral fractures in 16 patients presented with a typical history of acute back pain in an individual with osteoporosis [20]; however, substantial deformation of the vertebral body was not found on initial plain radiographs. Kanchiku et al. also reported that 10 out of 95 OVFs showing signal intensity changes on MRI were difficult to identify on plain radiographs because of almost complete lack of vertebral body collapse [12]. In the present study, 70.5% of the fractured vertebrae had anterior vertebral body height more than 80% of that expected in the first 2 weeks after injury, whereas the percentage decreased to 25.9% at 6 months. Along with the reports of Pham et al. [20] and Kanchiku et al. [12], the present results suggest that diagnosing acute fracture on X-ray is often difficult because of the lack of deformity in the acute stage.

MRI is considered a more accurate tool for OVF diagnosis than plain X-ray. Kanchiku et al. reported a diagnostic rate of 98% for fractured vertebral bodies by MRI, which was higher than the 87% by plain radiography [12]. Several abnormal MRI findings have been identified as typical signs of OVF: fracture line as low-intensity signal band on T1 image [11], bone edema as signal intensity change on T1- and/or T2-weighted images [17,21], and fluid sign as focal or linear hyperintense change on T2-weighted or short T1 inversion recovery (STIR) image [13]. However, in most studies, the period from onset of injury until MRI examination was not uniform, and it remains unclear which abnormal findings indicate acute fracture. Although the chronological changes in abnormal findings have not been well studied, there have been a few anecdotal reports on this issue. Yamato et al. reported that the bone marrow intensity change on T1-weighted images restores earlier than that on T2-weighted images [22]. Cho et al. demonstrated that restoration of signal intensity was slow near the endplate or the center of the vertebra in some types of fracture [23]. Based on these reports, we focused on intensity changes in the posterior wall of the fractured vertebral body on T1-weighted MRI to differentiate acute OVF.

The present study revealed that 75% is the best AVHR cutoff value to distinguish acute fracture from old and that this in combination with intensity change in the posterior wall on T1-weighted MRI is a good indicator of acute fracture. If a vertebral fracture is identified but the duration of the injury is uncertain, this combination of findings will be helpful to estimate the age of the fracture. However, the sensitivity and specificity of the abnormal intensity in the posterior wall on MRI were not high in diagnosing non-union, so normalization of intensity change in the posterior wall may not always indicate fracture healing. Further study including more detailed imaging analysis with longer follow-up will be necessary to identify the MRI findings that strongly support a diagnosis of non-union.

There are several limitations to note in this study. First, we only included patients whose date of fracture could be identified by the onset of pain or a specific injury. Therefore, these data may not be applicable for fractures without pain. Second, to simplify the study, we only evaluated AVHR on X-ray and posterior wall intensity change on T1-weighted MRI in the first 2 weeks after injury and 6 months after injury. Some vertebral fractures exhibit middle or posterior vertebral collapse without anterior collapse; in these cases, it may be more suitable to measure the middle or posterior vertebral height ratios rather than AVHR. Further studies that include middle/posterior vertebral height ratios, T2-weighted images, and STIR images at more time points will provide more accurate discriminators for the diagnosis of fracture age and pseudarthrosis. Finally, we have to note that the sensitivity and specificity in this study are for discrimination between acute and older fractures, and not for differentiating acute vertebral fracture from other pathological processes. For the appropriate diagnosis of acute OVF, it is important to look for the presence of other abnormal findings on imaging studies that may indicate neoplastic vertebral fracture or osteomyelitis [11,16,17].

## Conclusions

The AVHR was high in the acute stage within 2 weeks of injury but significantly decreased by 6 months. On MRI, 42% of the fractured vertebrae with low signal intensity change in the posterior wall during the acute stage had normalized by 6 months after injury. The combination of intensity change in the posterior wall on MRI and AVHR >75% on X-ray indicates a high probability of acute fracture.

## Abbreviations

OVF: osteoporotic vertebral fractures
MRI: magnetic resonance imaging
STIR: short T1 inversion recovery
AVHR: anterior vertebral height ratio
ROC: receiver operating characteristic
PPV: positive predictive value
NPV: negative predictive value
